# Malaria-MOI: A flexible and scalable tool for predicting multiplicity of infection in malaria parasites

**DOI:** 10.1186/s13073-026-01600-6

**Published:** 2026-01-22

**Authors:** Nina Billows, Jody Phelan, Joseph Thorpe, Leen N. Vanheer, Mark KI Tan, Susana Campino, Taane G. Clark

**Affiliations:** 1 Department of Infection Biology, Faculty of Infectious and Tropical Diseases, London School of Hygiene and Tropical Medicine, Keppel Street, London, WC1E 7HT UK; 2 Department of Infectious Diseases, King’s College London, London, UK; 3 Centre for Clinical Infection and Diagnostics Research, Guy’s and St. Thomas’ NHS Hospital, London, SE1 7EH UK; 4 Faculty of Epidemiology and Population Health, London School of Hygiene and Tropical Medicine, Keppel Street, London, WC1E 7HT UK

**Keywords:** Multiplicity of infection, Malaria, Plasmodium, Complexity of infection, Bioinformatic software

## Abstract

**Background:**

Estimating the multiplicity of infection (MOI) is critical for understanding malaria transmission dynamics and within-host *Plasmodium* parasite diversity. We developed Malaria-MOI, a fast, flexible, and species-agnostic Python tool that infers MOI directly from standard genomics files (e.g., BAM, VCF format) derived from whole genome sequencing (WGS), without requiring prior training data, curated panels or complex parameter tuning.

**Results:**

When benchmarked on 27 *Plasmodium falciparum* mixed-clone samples with known MOI, Malaria-MOI matched or outperformed leading methods, achieving a root mean squared error of 0.38, mean absolute error of 0.15, and a correlation of 0.78 using genomic variants across 165 diverse loci. These results exceeded the median performance of existing Bayesian and likelihood-based approaches. Applied to 8,208 *P. falciparum* field samples, Malaria-MOI showed a strong negative correlation with F_WS_ (ρ = − 0.76), consistent with its accurate capture of within-host diversity. It demonstrated high sensitivity for detecting monoclonal infections (0.99) and superior specificity (0.76) compared to estMOI (0.58), particularly at lower sequencing coverage. The tool also identified regional MOI differences aligned with transmission intensity, detecting more polyclonal infections in high-transmission areas such as West Africa, where estMOI underestimated complexity.

**Conclusions:**

Overall, Malaria-MOI accommodates diverse input types, including whole-genome data and diversity loci, and is compatible with both Illumina and Nanopore sequencing platforms. It is integrated into the Malaria-Profiler framework and is well-suited for genomic surveillance of *Plasmodium* and other pathogens, especially when elucidating transmission intensity for disease elimination activities.

**Software:**

https://github.com/LSHTMPathogenSeqLab/Malaria-MOI.

**Supplementary Information:**

The online version contains supplementary material available at 10.1186/s13073-026-01600-6.

## Background

Malaria is a life-threatening disease caused by *Plasmodium* parasites, with *P. falciparum* and *P. vivax* being the most prevalent and deadly species. In 2024, malaria accounted for an estimated 282 million cases and 610,000 deaths worldwide, with the highest burden falling on children under five in sub-Saharan Africa [1]. An important factor that can influence disease severity and transmission dynamics is multiplicity of infection (MOI), also known as complexity of infection (COI), which refers to the number of genetically distinct parasite strains infecting a single individual [2, 3]. High MOI is a valuable research indicator of transmission intensity and infection complexity, providing insights that could inform intervention strategies [4].

Genomic data play a crucial role in understanding malaria epidemiology, offering insights into parasite population structure, admixture and the genetic variation underlying transmission dynamics and key phenotypes such as drug resistance [5–8]. The presence of multiple genetically distinct strains within a single infection introduces significant complexity to genomic analyses. High MOI can confound estimates of population allele frequencies and inflate measures of heterozygosity, ultimately reducing the accuracy of studies focused on genetic diversity and evolutionary dynamics. To address this, researchers often prioritise monoclonal infections, excluding polyclonal infections based on laboratory assessments such as PCR genotyping of polymorphic loci (e.g., *msp1*,* msp2*, or *glurp*) or microsatellite analysis [9]. While this helps simplify downstream analysis, it also results in the loss of valuable genetic information and can misrepresent the true diversity of the parasite population, especially in high-transmission settings. This filtering may mask important signals associated with transmission dynamics, leading to an incomplete or diluted picture of malaria epidemiology. However, accurately estimating MOI remains challenging using whole-genome sequence (WGS) data. Single-nucleotide polymorphism (SNP) based methods are often limited by their low sensitivity to detect minority clones and WGS coverage is considered insufficient for confident MOI estimation. In contrast, deep sequencing of highly diverse amplicons (> 10,000-fold coverage) has become the gold standard for detecting low-frequency clones and estimating MOI, but with an increased need for complex computational approaches [10, 11]. Nonetheless, it remains important to identify polyclonal infections within WGS datasets, as this influences decisions about which samples to include in downstream analyses and ensures that the full extent of parasite diversity is accurately represented.

Genomic MOI tools leveraging genome-wide SNPs or targeted deep sequencing of informative loci can detect subtle genetic differences between co-infecting strains, quantify the number of distinct parasite lineages within an infection, and in some cases, infer their proportions or relatedness. These tools provide more accurate MOI estimates, enhancing downstream analyses of population structure, selection, drug resistance and transmission dynamics, particularly in high-transmission settings where polyclonal infections are common and can obscure genetic signals.

The estimation of MOI in malaria has advanced significantly in recent years, evolving from simple heuristic approaches to sophisticated statistical inference methods. One of the earliest and most widely used metrics is the F_WS_ statistic, implemented in tools such as pfmix and moimix [12]. The F_WS_ statistic, defined as $\:{F}_{WS}=1-{H}_{W}/{H}_{S}$, measures within-host parasite diversity relative to the population, where $\:{H}_{W}$is the within-sample heterozygosity (proportion of heterozygous SNPs within an infection) and $\:{H}_{S}$is the population-level heterozygosity at the same SNPs; values near 1 indicate mostly clonal infections, while values near 0 indicate high within-host diversity [12]. F_WS_, while simpler to compute and useful in surveillance, is sensitive to allele frequency distributions and should be interpreted alongside direct MOI estimates. Initial MOI estimation tools, including estMOI and COIL, employed heuristic or mixture model approaches using allele counts, with COIL specifically leveraging the binomial distribution to estimate the likelihood of COI levels based on the prevalence of monomorphic or polymorphic genotypes within each sample [13, 14]. Later, Bayesian frameworks like THE REAL McCOIL were developed, which jointly estimate MOI and population allele frequencies using Markov chain Monte Carlo (MCMC) sampling [15]. Such Bayesian methods incorporate prior distributions on parameters including MOI, allele frequencies and genotyping error rates, requiring informative datasets with multiple SNPs and infections. They model loci as independent and typically assume unrelated clones, enabling the quantification of uncertainty but often at a higher computational cost. Other tools such as pfmix and moimix implement Bayesian mixture models with reversible jump MCMC to infer clone proportions and MOI per infection, while MOIRE extends these approaches to multi-allelic data and incorporates relatedness among clones [14, 16]. The SNP-slice tool offers a Bayesian nonparametric method that reconstructs parasite sequences and assigns them to infections jointly, allowing for flexible modelling without limiting the number of loci [17]. Dcifer employs a naive estimation approach that first ranks loci within a sample by the number of detected alleles, then uses a locus of a specified rank (lrank) to estimate the MOI [18]. In contrast, DEploid leverages haplotype structure from a reference panel of clonal isolates as prior information to jointly infer the number of strains, their relative proportions and the haplotypes present within a mixed infection [19, 20]. DEploid applies an MCMC framework to accurately resolve within-sample allele frequency imbalances and reconstruct individual haplotypes, allowing detailed insight into malaria infections.

While many existing approaches perform well in estimating the MOI and haplotype structure, their applicability is often limited by several factors. These methods typically require additional informative inputs, such as high-quality reference panels, reliable priors, or assumptions about allele independence and genotyping error rates. Moreover, computational complexity, particularly for Bayesian methods using MCMC, and sensitivity to data quality can restrict both scalability and accuracy across diverse epidemiological settings. These limitations underscore the need for flexible, scalable tools that offer accurate MOI estimation while remaining practical for routine genomic analysis. To address this need, we developed Malaria-MOI, a Python-based heuristic tool that builds on the estMOI concept. Malaria-MOI estimates MOI directly from allele count data from WGS data without the need for reference panels or computationally intensive processes. It supports both biallelic and multiallelic markers and is designed for seamless integration into genomic surveillance platforms such as Malaria-Profiler [21]. By balancing robustness and scalability, Malaria-MOI offers a practical solution for MOI estimation to aid malaria control efforts.

## Implementation

### Implementation of the Malaria-MOI heuristic for MOI estimation

Malaria-MOI is a Python-based method that analyses sequencing reads aligned to single-nucleotide polymorphisms (SNPs) from BAM and VCF files (Table 1). For each SNP in the VCF, nearby variants within a maximum distance δ (default: 500 bp) are grouped into triplets {p₁, p₂, p₃} such that (p₃ - p₁) ≤ δ. For each read overlapping all three positions, the corresponding alleles h = (a₁, a₂, a₃) are extracted to form a haplotype, where each a_i_ ∈ {A, T, C, G}. Reads are filtered based on mapping quality (Q ≥ 10) and base quality (q ≥ 12). A frequency count f(h) is generated for each unique haplotype h, and the total number of valid reads at the site is denoted as: N = Σ f(h). To exclude noise, only haplotypes with support f(h) > max(c_min, φ × N) are retained, where c_min is a user-defined minimum count (default: 10) and φ is a minimum fraction threshold (default: 0.1). The number of such filtered haplotypes per triplet is computed across the genome, resulting in a list {n₁, n₂, ., n_k_}, where n_i_ is the number of distinct haplotypes for triplet i. The MOI is estimated as the 90th percentile of this distribution: MOI_est = P₉₀({n_i_}). This percentile-based approach captures the upper-bound of haplotype diversity across genomic regions, reflecting the number of distinct co-infecting parasite strains. Malaria-MOI is a command-line tool, has been implemented in Malaria-Profiler [21], and is available via a dedicated GitHub repository: https://github.com/LSHTMPathogenSeqLab/Malaria-MOI.

**Table 1:** Malaria-MOI inputs and Command-Line options

*Option*	*Required*	*Description*	*Example*
-h, --help	No	Display the help message and exit.	-h
--bam BAM	Yes	Input alignment file in BAM or CRAM format. Must be indexed (.bai or .crai file). It can be derived from Illumina or Oxford Nanopore Technologies (ONT) sequence data.	--bam sample.bam
--vcf VCF	Yes	Input VCF file containing variant (SNP) positions of interest. It can be derived from Illumina or ONT sequence data.	--vcf variants.vcf.gz
--outfile OUTFILE	Yes	Output filename for the results table.	--outfile output.txt
--maxdist MAXDIST	No	Maximum allowed distance (in base pairs) between the first and last SNP within a haplotype block.	--maxdist 1000
--min_count MIN_COUNT	No	Minimum count of haplotype.	--min_count 5

### Testing and validation for malaria MOI estimation

To test, validate, and evaluate the performance of Malaria-MOI, we used 27 *P. falciparum* samples with known mixed-clone proportions derived from four laboratory clones: 3D7, 7G8, HB3, and Dd2, available from the Pf3k dataset [19, 22, 23]. To estimate read mapping ratios, a combined reference FASTA file was created using high-quality assemblies 3D7, 7G8, HB3, and Dd2, and reads underwent competitive mapping using bowtie2 (v2.5.4) (--very-sensitive -k 10) [24, 25]. For MOI predictions raw FASTQ files were processed using the fastq2matrix pipeline [26]: reads were trimmed with Trimmomatic (v0.39), aligned to the reference genome using BWA-MEM (v0.7.17-r1188), and variants were called using GATK HaplotypeCaller (v4.1.4.1-1) with base quality score recalibration (BQSR) applied to BAM files [27–29]. Variant filtering followed GATK Best Practices using the following criteria: QD < 2.0, QUAL < 30.0, SOR > 3.0, FS > 60.0, MQ < 40.0, MQRankSum < -12.5 and ReadPosRankSum < -8.0, implemented via GATK VariantFiltration [27–29]. Malaria-MOI was benchmarked against several existing MOI estimation tools representing a range of methodological approaches, including heuristic, Bayesian, and likelihood-based estimators. Benchmarking was performed across two input configurations: [1] WGS data (used by Malaria-MOI and DEploid); [2] SNPs derived from 165 diverse loci (Mad^4^Hatter loci) (used by THE REAL McCOIL, SNP-slice, coiaf, MOIRE, Dcifer and Malaria-MOI) [15–19, 30, 31]. For tools requiring bi-allelic input, VCF inputs were filtered further to select bi-allelic SNPs. All tools were implemented using recommended and default settings with minor adjustments to improve tuning for more effective MOI prediction.

For SNP-slice [17], we modified the default parameters to better capture the biological variability present in mixed-clone infections derived from laboratory clone mixtures. Specifically, we selected a negative binomial model (model = 3) to account for overdispersion in allele count data. The prior shape parameter alpha was reduced from 2.6 to 0.8 to allow for greater flexibility in modelling variability, reflecting the complex mixture patterns observed in these controlled samples. A lower alpha increases the dispersion allowed by the prior, which improves model fit and MOI estimation accuracy when working with heterogeneous clone proportions typical of laboratory mixtures. Additional MCMC parameters were adjusted by increasing the burn-in to 5,000 iterations and enabling early termination when the posterior likelihood stabilised (gap = 500), thus enhancing convergence and robustness of the posterior MOI estimates.

For THE REAL McCOIL [15], parameter settings were tailored for SNP matrix input. The maximum COI (maxCOI) was set to 4, with thresholds for sample inclusion (threshold_ind) and locus inclusion (threshold_site) both set to 20, ensuring sufficient data coverage. The MCMC sampler was run for a total of 10,000 iterations (totalrun), including a burn-in period of 1,000 iterations (burnin). The initial COI (M0) was set to 1. Error rates for genotype calls were controlled via parameters e1 and e2, both set to 0.05, representing the probabilities of calling homozygous loci heterozygous and vice versa. The error model parameter (err_method) was set to 3, enabling joint estimation of error rates alongside COI and allele frequencies.

For comparisons to MOIRE [16], we modified the default settings to better suit the laboratory-controlled clone mixtures. We disabled relatedness inference (allow_relatedness = FALSE), reflecting the assumption of independent infections typical of known clonal lab mixtures and consequently omitted the relatedness priors (r_alpha = 1, r_beta = 1). Other parameters remained consistent with the default MOIRE settings: 10,000 MCMC samples (num_samples = 1e4) with a burn-in of 5,000 iterations (burnin = 5e3), and 40 parallel tempering chains (pt_chains = 40) to balance effective posterior exploration with computational efficiency. Thinning was set to 2. Error model priors were kept at default values. COI estimates were summarized using both summarize_coi() and summarize_effective_coi() functions. In addition, prior to running MOIRE, the multi-sample VCF file was filtered to retain only SNPs with a sequencing depth greater than 10, consistent with the recommended preprocessing workflow to ensure reliable variant calls.

We ran DEploid [19] with and without a reference haplotype panel, specifying a maximum of four strains (-k 4) and providing population allele frequencies via a PLAF file. For the panel-free run, we included the -noPanel and -ibd flags; for the panel-based run, we used a filtered panel of lab strains derived from Pf3k data [19], originally used in the DEploid evaluation to represent known clonal haplotypes.

In addition, we estimated COI using the coaif package [31] by running optimize_coi() with data_type = “real”, a maximum COI of 4 (max_coi = 4) and a sequencing error rate of 0.01 (seq_error = 0.01). These settings reflect the use of real biallelic SNP data with a modest error rate, appropriate for high-quality laboratory-generated sequence data. Dcifer was run with default settings and lrank of 2 [18].

Performance was evaluated by comparing estimated MOI values to known truth sets derived from experimental clone mixtures, using Pearson correlation, root mean squared error (RMSE), and mean absolute error (MAE) as evaluation metrics.

Furthermore, using the 27 *P. falciparum* mixed-clone samples with known MOI, we conducted a local sensitivity analysis. Each parameter, including percentile threshold (0.85, 0.9, 0.95), minimum haplotype fraction (--min_frac; 0.05, 0.1, 0.25), and minimum read count (--min_count; 5, 10) was varied individually while keeping the other parameters at their default values. This allowed us to assess the robustness of haplotype detection and MOI estimation across different parameter settings. We also measured the runtime and memory requirements for processing individual samples, as well as for batches of 100 and 1,000 samples, with 5 samples run in parallel.

### Application of Malaria-MOI and F_WS_ to global *P. falciparum* data

After benchmarking Malaria-MOI against other tools, we applied it to a globally diverse dataset of *P. falciparum* isolates. We randomly selected 8,208 isolates for downstream analysis (Additional file 1: Table S1). These samples were sourced from the MalariaGEN Pf7 open dataset and other public data, and 92.7% had undergone selective whole genome amplification (sWGA) [32–34]. Of these, 572 were replicate runs (207 unique samples), used to test for concordance. All samples were pre-processed using the fastq2matrix pipeline. Raw reads were trimmed using Trimmomatic (v0.39), aligned to the *P. falciparum* Pf3D7v3 reference genome using BWA-MEM (v0.7.17-r1188) and underwent base quality score recalibration (BQSR) using GATK (v4.1.4.1), with the *P. falciparum* Genetic Crosses 1.0 dataset used to model systematic basecall error [27–29]. Variants were called using GATK HaplotypeCaller in GVCF mode. In addition, to demonstrate the interoperability of Malaria-MOI, we applied our tool to ONT data using default settings for 6 *P. falciparum* field samples from Papua New Guinea [34]. Raw reads underwent standard quality checks as above, were mapped using minimap2 (v2.28-r1209), and variants were called using Freebayes (v1.3.8) as the basecalling model was not available [35].

To support Malaria-MOI analysis, individual VCFs were filtered directly using hard-filtering parameters consistent with GATK and ONT best practices, including thresholds on QD, FS, SOR, MQ, and other metrics. These filtered, single-sample VCFs were used to extract genotypes at 165 known diversity loci (Additional file 1: Table S2) [30].

For F_WS_ estimation, GVCF files were first imported into a GenomicsDB using GATK GenomicsDBImport (v4.1.4.1), followed by joint genotyping with GATK GenotypeGVCFs to generate a multi-sample VCF. This file underwent further processing using GATK’s VariantRecalibrator and ApplyVQSR (parameters: -an QD -an FS -an SOR -an DP -maxGaussians 8, --truth-sensitivity-filter-level 99.0), again using the *P. falciparum* Genetic Crosses 1.0 dataset [28]. Only SNPs with a VQSLOD > 0 were retained as high-confidence variants for use in F_WS_ computation. F_WS_ scores were calculated over genome-wide bi-allelic SNPs using the moimix (v0.0.2.9001) package in R [12]. This method compares within-host diversity to heterozygosity in a local population. Therefore, F_WS_ scores were calculated according to each region represented in the dataset and a MAF threshold of 0.01. This resulted in a value between 0 and 1. Values closer to 1 are used to indicate monoclonality, whereas values closer to 0 typically indicate polyclonal samples. A threshold of F_WS_ ≥ 0.95 was used to label samples as monoclonal. Any sample with a F_WS_ score less than this threshold was labelled as polyclonal to compare with Malaria-MOI estimates and calculate sensitivity and specificity scores. Sensitivity was defined as the proportion of true positive samples correctly classified as monoclonal by the tool, according to the F_WS_ reference classification.

Additional filtering steps were applied across both pipelines: samples with more than 40% missing genotype data were excluded. Samples were retained only if  ≥ 60% of both the whole genome and the 165 diversity loci were covered at a read depth of at least 5. This threshold served as a minimum inclusion criterion to exclude low-quality samples. The average coverage across retained samples was substantially higher across the 165 diversity regions (median depth across 165 diversity loci of 72.62-fold). Detailed per-sample coverage statistics are provided in Additional file 1: Table S1. Coverage was quantified using mosdepth (v0.3.8) in 500 bp windows. Samples were stratified by sequencing depth: low (median depth ≤ 10-fold), medium (10-fold < median depth ≤ 20-fold), and high (median depth > 20-fold).

## Results

### Benchmarking Malaria-MOI against established MOI estimation tools

**Fig. 1:**
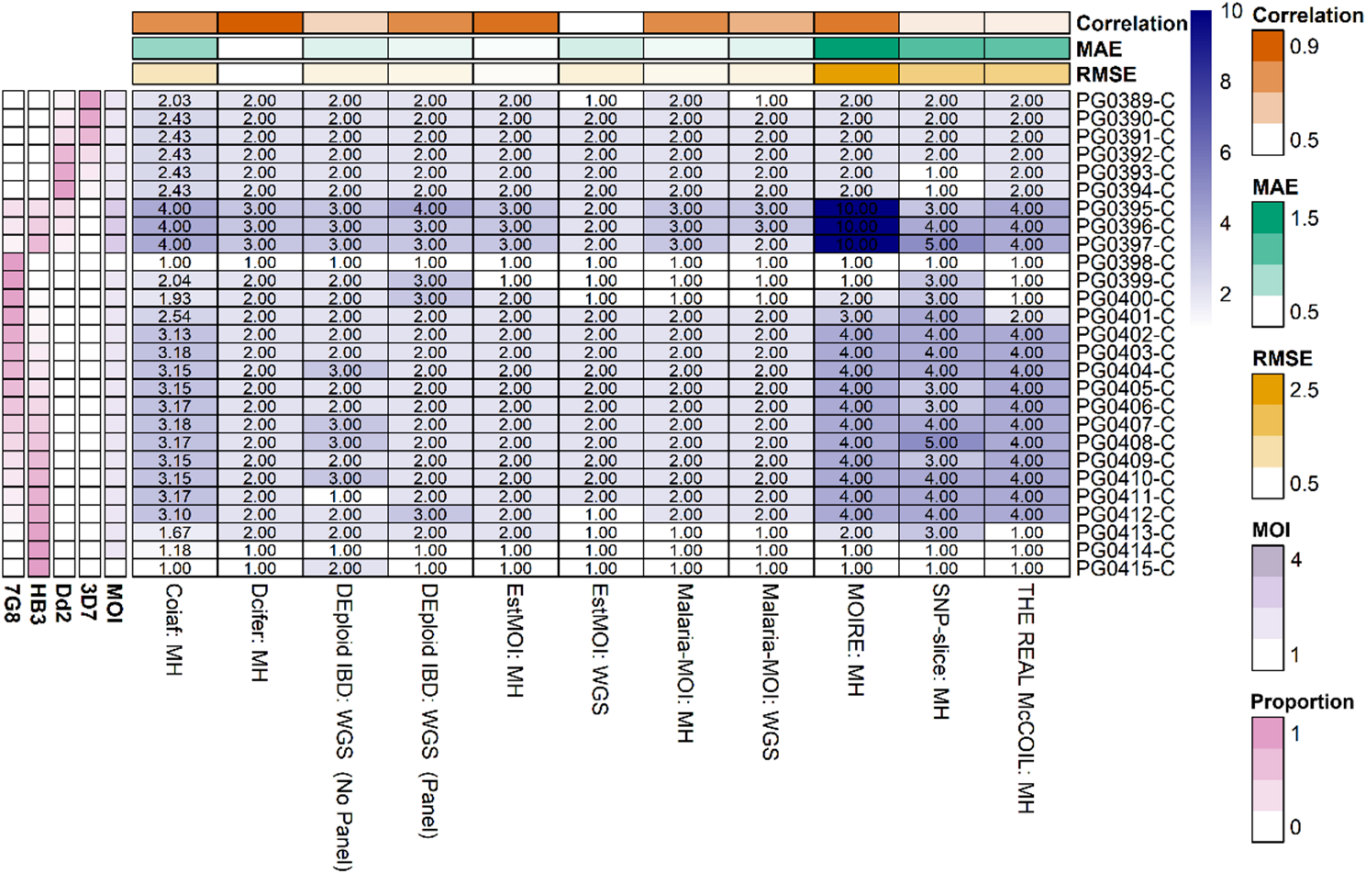
Comparison of performance of Malaria-MOI with other MOI estimation tools Heatmap shows predicted MOI values for 27 laboratory mixtures containing known proportions of *P. falciparum* clones (3D7, HB3, 7G8, Dd2; Table 2), using either SNPs from across the whole genome or a panel of diverse loci (diversity loci, MH) as input. Each row corresponds to a mixture; the true MOI is indicated alongside for reference. Tools evaluated include Malaria-MOI, estMOI, coiaf, THE REAL McCOIL, SNP-slice, Dcifer, DEploid (IBD) and MOIRE. Summary metrics in the top panel show root mean square error (RMSE), mean absolute error (MAE), and Pearson correlation between predicted and true MOI values

Using 27 laboratory mixtures of known proportions of the *P. falciparum* clones 3D7, Dd2, 7G8, and HB3, we compared the performance of Malaria-MOI against several established MOI estimation tools [19, 22, 23]. Based on root mean square error (RMSE), mean absolute error (MAE) and correlation, Malaria-MOI performed comparably to existing tools, particularly when using a panel of diverse loci [30]. With diversity loci input, Malaria-MOI achieved an RMSE of 0.38, MAE of 0.15, and correlation of 0.78 (Fig. 1). This was notably better than the median RMSE (0.51), MAE (0.26) and correlation (0.77) across most other tools tested. Malaria-MOI demonstrated substantial performance gains when using carefully selected, diverse loci compared to SNPs across the whole genome. This likely reflects the advantage of using highly informative, polymorphic loci that are curated to maximise the within-host diversity signal, whereas whole genome SNPs often include many invariant or low-frequency sites that introduce noise and reduce resolution for detecting multiple clones. The laboratory mixture panel included clone ratios ranging from 90:10 to 99:1 (Table 2). Despite minor ambiguity between closely related American strains HB3 and 7G8 in some samples, read mapping ratios generally supported the expected laboratory mixture ratios (Table 2). Malaria-MOI accurately detected minor clones present at frequencies as low as ~ 1%, indicating high sensitivity to low-frequency variants under the sequencing depths used. As with other methods, performance declined when minor strain proportions were very low (< 5%), but overall, MOI estimates remained stable and consistent (Fig. 1). Notably, the performance of Bayesian MCMC-based tools such as MOIRE and THE REAL McCOIL appeared sensitive to parameter choices (e.g., maximum COI) and prior settings, suggesting that further optimisation could potentially improve their accuracy. Their performance may also be optimised using amplicon data.

**Table 2:** *Plasmodium falciparum* laboratory mixture ratios, competitive read mapping, and predicted multiplicity of infection (MOI) using Malaria-MOI

Sample	Laboratory Mixture Ratios (Read Mapping Ratios)				Median Depth	Predicted MOI
	3D7	Dd2	HB3	7G8		
PG0389-C	0.9 (0.41)	0.1 (0.21)	0 (0.19)	0 (0.19)	126	2
PG0390-C	0.8 (0.39)	0.2 (0.23)	0 (0.19)	0 (0.19)	137	2
PG0391-C	0.67 (0.35)	0.33 (0.26)	0 (0.19)	0 (0.19)	130	2
PG0392-C	0.33 (0.27)	0.67 (0.34)	0 (0.19)	0 (0.19)	126	2
PG0393-C	0.2 (0.24)	0.8 (0.37)	0 (0.19)	0 (0.20)	118	2
PG0394-C	0.1 (0.22)	0.9 (0.40)	0 (0.19)	0 (0.19)	125	2
PG0395-C	0 (0.19)	0.33 (0.28)	0.34 (0.27)	0.33 (0.26)	119	3
PG0396-C	0 (0.20)	0.25 (0.25)	0.5 (0.25)	0.25 (0.30)	106	3
PG0397-C	0 (0.20)	0.14 (0.23)	0.71 (0.23)	0.14 (0.34)	113	3
PG0398-C	0 (0.19)	0 (0.19)	0 (0.41)	1 (0.20)	122	1
PG0399-C	0 (0.20)	0 (0.20)	0.01 (0.40)	0.99 (0.21)	120	1
PG0400-C	0 (0.19)	0 (0.19)	0.05 (0.40)	0.95 (0.21)	113	1
PG0401-C	0 (0.20)	0 (0.20)	0.1 (0.39)	0.9 (0.22)	121	2
PG0402-C	0 (0.19)	0 (0.19)	0.15 (0.38)	0.85 (0.23)	136	2
PG0403-C	0 (0.19)	0 (0.19)	0.2 (0.37)	0.8 (0.24)	120	2
PG0404-C	0 (0.19)	0 (0.19)	0.25 (0.36)	0.75 (0.25)	115	2
PG0405-C	0 (0.19)	0 (0.19)	0.3 (0.35)	0.7 (0.26)	100	2
PG0406-C	0 (0.19)	0 (0.19)	0.4 (0.33)	0.6 (0.28)	112	2
PG0407-C	0 (0.19)	0 (0.19)	0.5 (0.31)	0.5 (0.30)	104	2
PG0408-C	0 (0.20)	0 (0.19)	0.6 (0.29)	0.4 (0.32)	107	2
PG0409-C	0 (0.20)	0 (0.19)	0.7 (0.27)	0.3 (0.34)	107	2
PG0410-C	0 (0.20)	0 (0.19)	0.75 (0.26)	0.25 (0.36)	105	2
PG0411-C	0 (0.20)	0 (0.19)	0.8 (0.25)	0.2 (0.36)	117	2
PG0412-C	0 (0.20)	0 (0.19)	0.85 (0.23)	0.15 (0.38)	97	2
PG0413-C	0 (0.20)	0 (0.20)	0.95 (0.21)	0.05 (0.40)	110	1
PG0414-C	0 (0.20)	0 (0.19)	0.99 (0.20)	0.01 (0.41)	109	1
PG0415-C	0 (0.20)	0 (0.20)	1 (0.20)	0 (0.41)	98	1

^a^Reads underwent competitive mapping to 3D7, Dd2, HB3 and 7G8 reference assemblies; ^b^Median depth when mapped to Pf3D7v3;^c^Malaria-MOI prediction using default settings

We also assessed how changes in Malaria-MOI parameters, including the percentile threshold (0.85, 0.9 and 0.95), minimum haplotype fraction (0.05, 0.1 and 0.25) and minimum read count (5 and 10) affected Malaria-MOI prediction for the 27 *P. falciparum* mixed clones. Changes to parameters led to minimal differences in MOI predictions made by Malaria-MOI. Only a single difference is reported for PG0397-C where a lower MOI of 2 was observed using a percentile threshold of 0.85 (Additional file 1: Table S3). This is because the MOI estimate is derived from the percentile of haplotype counts across all triplets. Reducing this threshold reduces the diversity of triplets used to estimate MOI, thus decreasing MOI.

### Global evaluation of infection complexity

We further demonstrate the application of Malaria-MOI to real-world data by investigating how MOI varies across different regional settings. Malaria-MOI was applied to a large dataset of 8,208 field *P. falciparum* samples from eight geographic regions: West Africa (*n* = 3,006), Southeast Asia (*n* = 2,636), South Asia (*n* = 1,388), East Africa (*n* = 755), South America (*n* = 124), Central Africa (*n* = 194), Oceania (*n* = 72) and the Horn of Africa (*n* = 33) (Additional file 1: Table S4). Estimates from Malaria-MOI were derived using highly diverse loci. In parallel, we calculated within-host diversity using the F_WS_ statistic for each sample as an established complementary measure of infection complexity. While F_WS_ reflects the relative heterozygosity of a sample in the context of population-level diversity, relying on background allele frequencies and sample grouping, Malaria-MOI estimates the number of clones directly from individual samples without requiring population-level comparisons. For additional context, we also included estMOI, which uses a similar methodology to Malaria-MOI, to enable direct comparison. Together, these three approaches were evaluated for consistency and to enhance our understanding of infection complexity across global regions. As ‘true MOI’ gold-standard values are not available for large-scale WGS datasets, we evaluate the sensitivity and specificity of Malaria-MOI and estMOI to detect monoclonal and polyclonal infections as defined using standard F_WS_ thresholds (Monoclonal: F_WS_ ≥ 0.95), which has previously been corroborated with MSP genotyping methods [12]. The proportion of polyclonal infections (F_WS_ < 0.95) varied by region, with the highest levels observed in Central Africa (59.8%), West Africa (52.0%), and East Africa (50.9%), intermediate levels in South Asia (38.5%) and South America (22.6%), and substantially lower proportions in Southeast Asia (18.9%), the Horn of Africa (15.2%) and Oceania (9.0%). Sensitivity was defined as the proportion of true positive samples that were correctly identified as monoclonal by the tool, based on the F_WS_ classification. This definition was selected because accurately identifying monoclonal infections is critical for downstream analyses that assume single-clone infections.

Spearman correlation analyses showed a strong negative association between F_WS_ and MOI estimates from both Malaria-MOI (ρ = -0.76, *p* < 0.001) and estMOI (ρ = -0.66, *p* < 0.001) across 8,208 samples, consistent with the expectation that higher within-host diversity corresponds to higher MOI. Both Malaria-MOI and estMOI methods exhibited very high sensitivity of 0.99, indicating their ability to detect monoclonal infections (Table 3). However, Malaria-MOI demonstrated higher specificity than estMOI (0.76 vs. 0.58), suggesting it is better at correctly identifying polyclonal infections and reducing false positives. This difference may reflect Malaria-MOI’s improved resolution or tuning in distinguishing low-diversity infections, whereas estMOI may be more prone to overestimating complexity in near-clonal samples. It should also be noted performance metrics could be less reliable for samples with diversity near the selected monoclonal/polyclonal threshold of 0.95. In such cases, small fluctuations in allele frequencies or sequencing noise may lead to ambiguous classification, lowering specificity.

**Table 3:** Sensitivity and specificity of estmoi and Malaria-MOI to predict monoclonal vs. Polyclonal P. falciparum infections

	Sensitivity				Specificity			
	Overall	Low Coverage^a^	Medium Coverage^b^	High Coverage^c^	Overall	Low Coverage^a^	Medium Coverage^b^	High Coverage^c^
EstMOI	0.99	1.00	0.98	0.99	0.58	0.36	0.44	0.61
Malaria-MOI	0.99	0.95	0.97	1.00	0.76	0.72	0.65	0.77

^a^Low: median depth ≤ 10-fold; ^b^Medium :10-fold < median depth ≤ 20-fold; and ^c^High: median depth > 20-fold

The runtime and memory usage for Malaria-MOI across global samples were also recorded. The mean runtime was 24.3 s per sample (range: 19.5–41.5 s), with an average memory usage of 38.7 MB (maximum 42 MB). During benchmarking, up to 5 samples were run in parallel. Based on these measurements, the estimated wall-clock runtime for processing 100 samples with 5 samples in parallel is approximately 8.1 min, and for 1000 samples it is approximately 81.2 min. This highlights the scalability of Malaria-MOI for analysing large-scale WGS data.

### Influence of sequencing coverage on Malaria-MOI estimates and discordance analysis

To evaluate the impact of sequencing coverage on method performance, we assessed the sensitivity and specificity of estMOI and Malaria-MOI in classifying infections as monoclonal or polyclonal across low (median depth ≤ 10-fold), medium (10-fold < median depth ≤ 20-fold), and high coverage settings (median depth > 20-fold), using F_WS_ as an indicator of clonality. Sensitivity remained consistently high for both methods across all coverage levels, indicating robust detection of monoclonal infections. The estMOI approach showed minimal variation, with sensitivity ranging from 0.98 to 1.00, while Malaria-MOI ranged from 0.95 at low coverage to 1.00 at high coverage (Table 3). In contrast, specificity, the ability to correctly identify polyclonal infections, was more strongly influenced by coverage depth. EstMOI exhibited low and variable specificity, particularly at low (0.36) and medium (0.44) coverage, with moderate improvement at high coverage (0.61). Malaria-MOI demonstrated higher overall specificity and a clearer coverage dependence, with values of 0.72 at low, 0.65 at medium, and 0.77 at high coverage (Table 3). These results suggest that while sensitivity is largely unaffected by sequencing depth, the ability to accurately detect polyclonal infections, especially for estMOI, is more sensitive to data quality, with higher coverage improving classification performance.

Further assessment of discordant results between estMOI, Malaria-MOI and F_WS_ highlighted that specificity was also likely to have been influenced by the threshold used to indicate monoclonal infections (F_WS_ ≥ 0.95) (Additional file 2: Fig. S1 and Fig S2.). Most discordant results had F_WS_ close to the 0.95 threshold, where 54% of discordant polyclonal infections (defined by F_WS_<0.95) had F_WS_ scores greater than 0.90. Examples of samples that were classified as polyclonal with F_WS_ <0.80 were rarer (< 1% of total samples) and could be due to additional heterozygosity outside of the 165 diversity regions utilised. Samples predicted to be monoclonal by Malaria-MOI but predicted as polyclonal using F_WS_ were infrequent (< 1% of total samples). This is likely due to similar or homogenous clones being present in the sample that can be more easily distinguished using diverse regions over WGS data. Similar observations were made for comparisons between estMOI and F_WS_ (Additional file 2: Fig. S2). A comparison of sample-level estimates of estMOI and Malaria-MOI revealed that 13.69% of samples had discordant results. Of these, 63% of samples were predicted to be polyclonal by Malaria-MOI but monoclonal by estMOI, 96% of which matched F_WS_ definitions of polyclonality (Additional file 2: Fig. S2). This highlights a greater capability of Malaria-MOI to detect polyclonal samples from genomic data over estMOI and is consistent with the greater specificity of Malaria-MOI for predicting monoclonal infections (Table 3). Finally, we also assessed discordance amongst 572 replicate isolates (207 samples). Of these, Malaria-MOI had generally consistent results with only 9 samples being predicted inconsistently classified as polyclonal. This result was consistent with F_WS_ estimation. This could be due to small fluctuations in read depth or allele frequency between replicates or sampling heterogeneity affecting the representation of minor clones.

### Regional variation in infection complexity across global *P. falciparum* samples

**Fig. 2:**
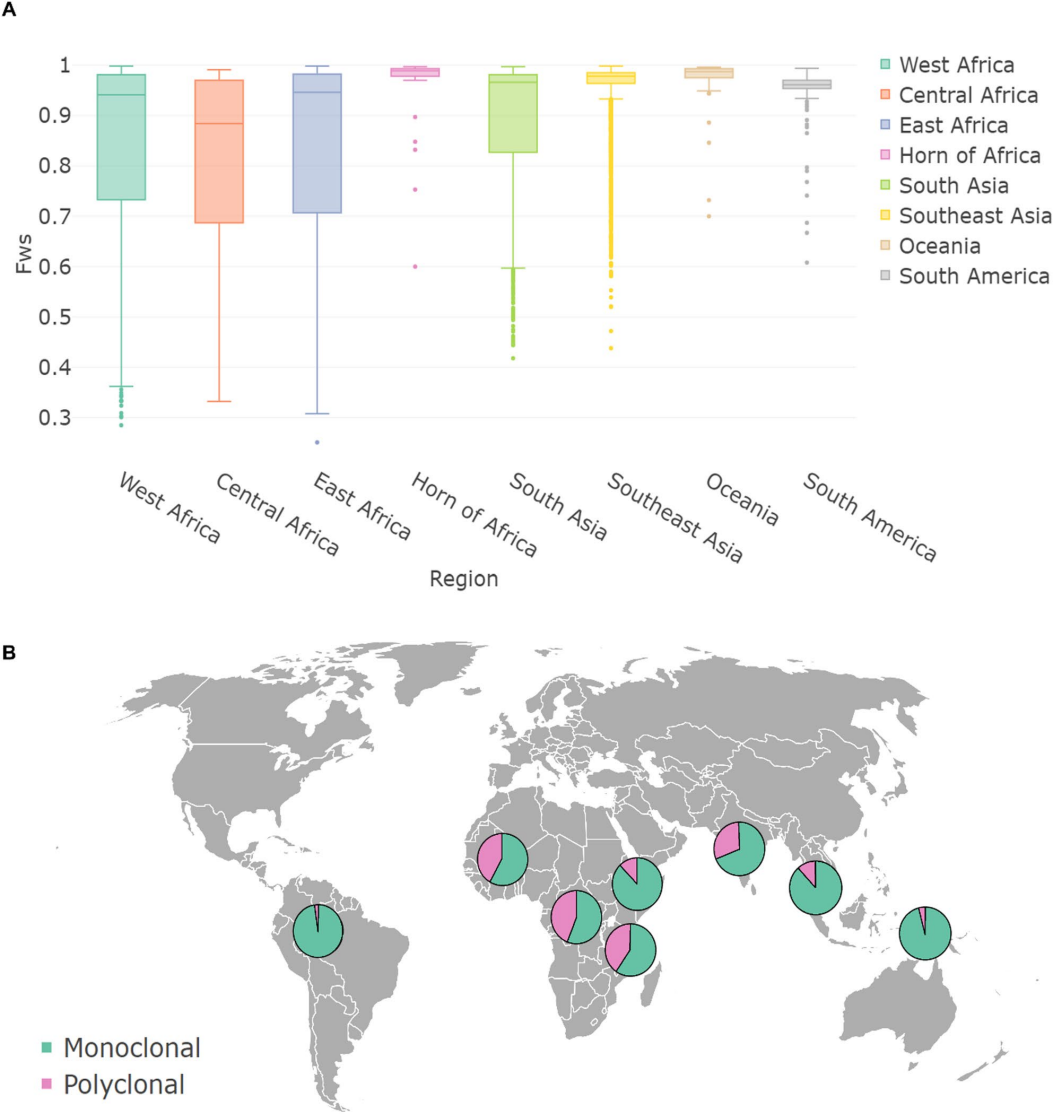
Geographic distribution of monoclonal and polyclonal *P. falciparum* infections across eight regions (**A**) Boxplots showing the distribution of F_WS_ scores by region, reflecting within-host diversity. Regions include West Africa (*n* = 3,006), Southeast Asia (*n* = 2,636), South Asia (*n* = 1,388), East Africa (*n* = 755), Central Africa (*n* = 194), South America (*n* = 124), Oceania (*n* = 72), and the Horn of Africa (*n* = 33). (**B**) Proportion of samples classified as monoclonal or polyclonal by Malaria-MOI in each region. Pie charts display the percentage of samples in each category

Across global *P. falciparum* samples, estMOI and Malaria-MOI provide broadly consistent estimates of infection complexity, but notable differences emerge in how they classify MOI categories across regions (Fig. 2). Malaria-MOI consistently detects a higher proportion of polyclonal infections, particularly in high-transmission settings, suggesting greater sensitivity to mixed infections. For example, in West Africa, a region of intense malaria transmission, Malaria-MOI estimates that only 58% of infections are monoclonal (MOI = 1), compared to 69.5% with estMOI, with a corresponding increase in higher MOI classifications (MOI = 2 and MOI = 3) (Fig. 2). Similar patterns are observed in East and Central Africa, where Malaria-MOI identifies more infections with MOI > 1 than estMOI. In contrast, both methods report a high proportion of monoclonal infections in low-transmission regions such as Southeast Asia, Oceania and South America (Fig. 2). For instance, in South America, Malaria-MOI classifies 98.4% of samples as MOI = 1, aligning with expectations for settings with limited superinfection and lower parasite genetic diversity. This is further supported by low MOIs estimated using ONT data, where 6 samples from Papua New Guinea all had a predicted MOI of 1 (Additional file 1: Table S1). These regional patterns broadly reflect established epidemiological trends, with higher MOI in high-transmission regions due to frequent superinfection and recombination, and lower MOI in areas with more focal or controlled transmission. The increased sensitivity of Malaria-MOI to subtle within-host diversity makes it a particularly valuable tool for monitoring transmission intensity and assessing the impact of control interventions over time.

## Discussion

Whole-genome sequencing (WGS) of *Plasmodium* parasites has expanded rapidly, with global datasets now exceeding 20,000 samples for *P. falciparum* and 2,000 samples for *P. vivax* [32, 36]. Analyses of these large-scale datasets have provided key insights into drug resistance, population structure, genetic diversity and signals of selection. WGS has also become a valuable tool for genomic surveillance, supporting the development of platforms such as Malaria-Profiler, which can predict drug-resistance genotypes and infer the geographic origin of *Plasmodium* samples [21]. Multiplicity of infection (MOI) introduces additional complexity to genomic analyses. Traditionally, samples identified as polyclonal using laboratory methods were often excluded from sequencing to avoid analytical challenges. The development of MOI estimation tools has helped overcome this limitation by enabling MOI to be assessed directly from genome sequencing data, facilitating the inclusion of polyclonal infections in large-scale studies without requiring extensive resources. Early heuristic approaches, such as estMOI, provided a rapid means to identify polyclonal infections from genome sequences [13].

Additional development of more sophisticated tools, based on Bayesian, Markov Chain Monte Carlo (MCMC), and likelihood frameworks, such as COIL, SNP-slice, MOIRE, and THE REAL McCOIL, aimed to improve the accuracy of MOI estimation [15–17]. These approaches often incorporate additional information, such as the genetic relatedness between samples, to refine their predictions [15–17, 31]. Further innovations, including the use of multi-allelic SNPs (as in MOIRE and Dcifer) and haplotype-based approaches (such as DEploid), have also advanced MOI estimation. Although many of these tools perform well in practice, their integration into widely used genomic surveillance pipelines remains limited. For some existing tools, the need for extensive tuning of priors and parameters, reliance on population-level allele frequency (PLAF) estimates, the use of predefined SNP panels, and a focus on bi-allelic SNPs can limit their suitability for routine genomic monitoring of *Plasmodium* species. To address these challenges, we developed Malaria-MOI, a Python-based heuristic tool designed to offer a flexible, user-friendly solution for estimating MOI from individual samples without the need for complex model calibration.

The use of Bayesian approaches to estimate MOI, such as COIL, model the probability of observed allele counts given a hypothesised number of clones, often incorporating population-level allele frequencies as priors. While these methods are statistically robust and provide uncertainty estimates, they do not leverage haplotype linkage information within sequencing reads. This information is useful for estimating MOI as it captures which alleles co-occur on the same read, allowing accurate detection of multiple co-infecting clones. The haplotype-based approach employed by Malaria-MOI reduces dependence on population priors and enables accurate discrimination between monoclonal and polyclonal infections. By combining stringent quality filtering with read-level haplotype reconstruction, Malaria-MOI provides a robust and reproducible framework for MOI estimation in both individual samples and large-scale population genomic studies. Malaria-MOI also reconstructs haplotypes directly from raw BAM and VCF data, making it an easy and flexible tool to integrate into existing pipelines. This is further exemplified by the benchmarking results, which show that Malaria-MOI is computationally efficient and scales well, with 1,000 samples (5 in parallel) requiring approximately 81.2 min of wall-clock time and 193.5 MB of memory.

We initially evaluated the performance of Malaria-MOI by comparing its MOI estimates to the known ‘true’ MOI values from controlled mixtures of *P. falciparum* clones (3D7, 7G8, HB2, and Dd2) with defined proportions [19, 22, 23]. Malaria-MOI demonstrated comparable or superior performance to existing tools, underscoring that heuristic approaches can perform as well as, or even outperform, more complex methods. This is further supported by the strong performance of the naïve MOI estimation method implemented within Dcifer [18]. It is important to note that the performance of comparator tools may improve with additional parameter tuning and careful selection of priors. Malaria-MOI achieved its best performance when using diversity loci, leveraging multi-allelic SNPs across 165 diverse loci previously shown to be highly informative for MOI estimation, compared to using genome-wide SNP inputs [30]. Using multi-allelic SNPs across carefully selected loci offers distinct advantages for MOI estimation compared to whole-genome data.

Genome-wide SNP datasets are typically dominated by biallelic sites, many of which are uninformative and can introduce noise into the analysis. In contrast, diversity loci are specifically selected for their high diversity and informativeness, providing finer resolution for distinguishing within-host strain complexity [37]. By capturing multiple alleles per locus, diversity loci are particularly effective at detecting polyclonal infections, even at moderate sequencing depths. Using 165 carefully selected diverse loci rather than WGS improves MOI estimation because it concentrates on regions with the highest allelic diversity. By computing haplotype counts per triplet and taking the 90th percentile, the Malaria-MOI approach effectively captures the maximum number of distinct clones in a sample. In contrast, standard WGS often lacks the coverage to detect low-frequency variants across the genome, making minority clones difficult to resolve. Focusing on highly polymorphic loci, including many antigenic genes, therefore provides a stronger diversity signal and more accurate MOI estimates. Their targeted nature also reduces computational demands and processing time, making them well-suited for large-scale genomic analyses, an approach that could be further enhanced through amplicon sequencing.

Furthermore, Malaria-MOI was applied to a global *P. falciparum* dataset consisting of 8,208 sequences to assess infection complexity worldwide and identify regional variations [32]. Malaria-MOI estimates were categorised into monoclonal and polyclonal infections and compared with F_WS_, a widely used population genetics metric in malaria genomics that measures within-host diversity relative to the local parasite population diversity [12]. Malaria-MOI was also compared to estMOI, a Perl-based heuristic tool for MOI estimation. Both methods demonstrated strong capabilities in detecting monoclonal infections [13]. However, Malaria-MOI was found to be more specific, capturing a greater proportion of monoclonal and polyclonal samples. While a gold-standard laboratory-based approach would further improve the accuracy of these metrics, our results demonstrate the suitability of Malaria-MOI for estimating MOI both in individual samples and across large global datasets for population genomic analyses. We also show that both Malaria-MOI and estMOI reliably detect monoclonal infections across varying coverage levels. However, Malaria-MOI exhibits greater specificity and superior performance in identifying polyclonal infections, particularly at higher coverage, whereas estMOI shows lower and more variable specificity across coverage depths. This improved ability of Malaria-MOI to detect polyclonal infections likely stems from its refined filtering criteria and haplotype-based approach. By grouping nearby SNPs into microhaplotypes and applying stringent quality filters on both mapping and base quality, Malaria-MOI effectively reduces false signals caused by sequencing errors or low-frequency variants that could otherwise be misinterpreted as additional clones. This is a key improvement of Malaria-MOI compared to estMOI, which can underestimate MOI in polyclonal samples.

Finally, we demonstrate regional variation in MOI across global *P. falciparum* populations. These differences broadly correspond to transmission intensity, with higher MOI observed in regions like West, Central and East Africa, and lower MOI in areas such as Southeast Asia and South America [1]. This highlights that MOI can be useful indirectly to inform genomic surveillance, serving as an indicator for the level of ongoing transmission. While MOI can be associated with transmission, it is influenced by multiple factors, which can include host immunity, parasite genetics, sampling strategies and the sensitivity of detection methods [38–41]. Therefore, while MOI provides valuable insights into transmission patterns, interpreting it requires careful consideration of these biological and technical complexities.

Whilst we have demonstrated the utility of Malaria-MOI for MOI estimation, there are several areas of further study that could be of value. Firstly, a lack of ‘true MOI’ estimates accompanying WGS makes it challenging to benchmark MOI estimation using real world data. Here, we used F_WS_ as a proxy for the gold standard, as it has previously shown to corroborate with MSP genotyping [12]. However, it should be notes that F_WS_ may have limited resolution for distinguishing infections composed of highly related or genetically homogeneous clones. Future validation of MOI estimates, particularly those greater than five in high-transmission settings, could benefit from paired datasets that combine known MOI values determined by high-resolution amplicon sequencing and MSP genotyping with corresponding WGS data. Deeper (amplicon) sequencing data from Illumina or ONT platforms could enable the detection of low-frequency clones, but it is possible to overestimate MOI through the selection of a limited number of diverse loci used. Future algorithmic developments could optimise parameter settings for amplicon-based data and incorporate quality control steps, such as the detection and removal of sequencing errors and chimeric reads. Future applications of Malaria-MOI could include *P. vivax*, which presents unique challenges due to its frequent relapses and population structure. More broadly, reconstructing co-infecting strains could provide deeper insights into malaria transmission dynamics across *Plasmodium* species. However, such analyses are computationally intensive. In the absence of these advanced approaches, we present a rapid MOI estimation method that is well-suited for genomic surveillance of *Plasmodium* parasites and other pathogens, thereby supporting disease elimination efforts.

## Conclusions

In summary, we have developed Malaria-MOI, a flexible and robust tool for estimating MOI from WGS data. It demonstrates strong performance across a range of sample types and coverage levels, making it well-suited for varied genomic analysis contexts. While this study focused on *P. falciparum*, Malaria-MOI is also applicable to other *Plasmodium* species and organisms where MOI estimation is relevant, such as *Trypanosoma* species. The tool supports a wide range of input data types and is compatible with both short-read and long-read sequencing platforms, including Illumina and ONT, offering versatility across different surveillance settings. Moreover, Malaria-MOI is fully integrated into the Malaria-Profiler pipeline, enabling seamless incorporation into broader malaria genomic workflows [21].

## Supplementary Information

Additional file 1. Supplementary Figures S1-S2

Additional file 2. Supplementary Tables S1-S4

## Data Availability

No new samples were sequenced as part of this study. Code for Malaria-MOI can be found in a dedicated GitHub repository: https://github.com/LSHTMPathogenSeqLab/Malaria-MOI. Accessions for previously published samples can be found in Additional file 1 Table S1 and were obtained from the MalariaGEN Plasmodium falciparum Community Project as described in ‘An open dataset of Plasmodium falciparum genome variation in 7,000 worldwide samples. MalariaGEN et al, Wellcome Open Research 2021642 DOI: 10.12688/wellcomeopenres.16168.1’. Laboratory mixtures were produced by Wendler J. Accessing complex genomic variation in Plasmodium falciparum natural infection. Ph. D. thesis, University of Oxford. 2015. and obtained via Zhu SJ, Almagro-Garcia J, McVean G. Deconvolution of multiple infections in Plasmodium falciparum from high throughput sequencing data. Bioinformatics. 2018;34(1):9-15.
