# Supplementary material for: Malaria-MOI: A flexible and scalable tool for predicting multiplicity of infection in malaria parasites
Source: Genome Med. 2026 Jan 22;18:22. doi: 10.1186/s13073-026-01600-6 (PMC12910736; doi:10.1186/s13073-026-01600-6)
Supplement: Supplementary file 1 — Additional file 1. Supplementary Figures S1-S2 [file 13073_2026_1600_MOESM1_ESM.docx]

**Malaria-MOI: A Flexible and Scalable Tool for Predicting Multiplicity of Infection in Malaria Parasites**

Nina Billows^1,**^, Jody Phelan^1,**^, Joseph Thorpe^1^, Leen N. Vanheer^1^, Mark KI Tan^1,2,3^, Susana Campino^1,***^ and Taane G. Clark^1,4,*,***^

^1^Department of Infection Biology, Faculty of Infectious and Tropical Diseases, London School of Hygiene and Tropical Medicine, Keppel Street, London WC1E 7HT., ^2^Department of Infectious Diseases, King's College London, London, UK., ^3^Centre for Clinical Infection and Diagnostics Research, Guy's & St. Thomas' NHS., ^4^Faculty of Epidemiology and Population Health, London School of Hygiene and Tropical Medicine, Keppel Street, London WC1E 7HT

*To whom correspondence should be addressed.

**Joint first authors

*** Joint last authors

**Supplementary Figures**


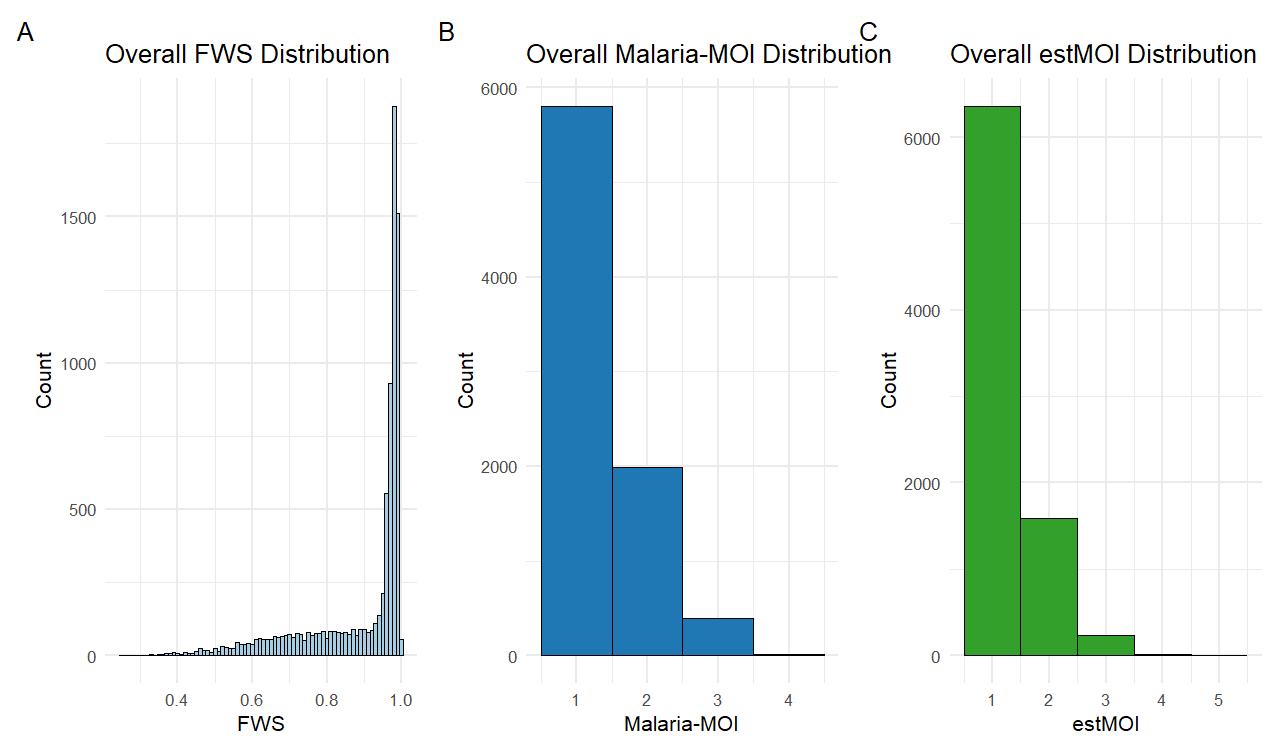


**Fig. S1. Overall distributions of F_WS_, Malaria-MOI, and estMOI.**
Panels A–C show the distributions of (A) F_WS_ values, (B) Malaria-MOI values and (C) estMOI values across all samples. Histograms depict the number of samples within each bin, illustrating the overall patterns of multiplicity of infection and within-host diversity in the study population.


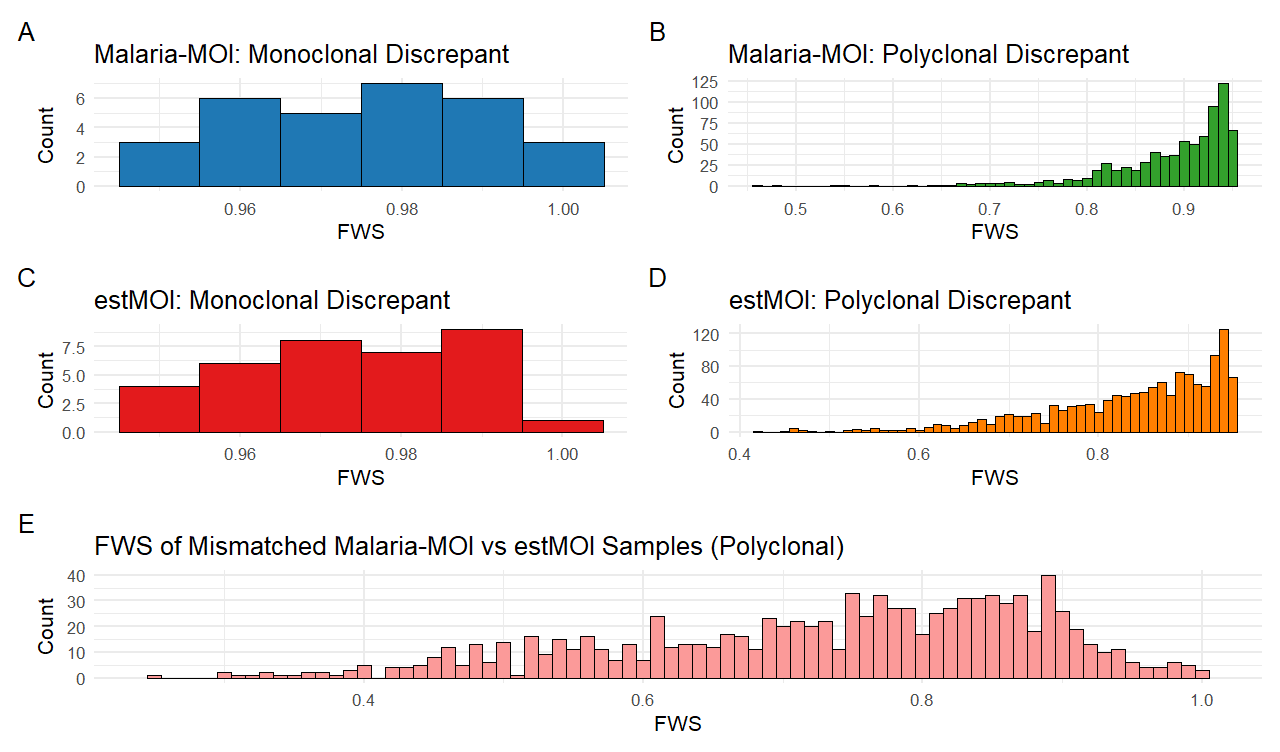


**Fig. S2. Discordance between Malaria-MOI, estMOI, and F_WS_.**
Panels A–E show distributions of FWS values among samples with classification discordance across the three metrics: (A) Malaria-MOI: Monoclonal samples with F_WS_ ≥ 0.95; (B) Malaria-MOI: Polyclonal samples with F_WS_ < 0.95; (C) estMOI: Monoclonal samples with F_WS_ ≥ 0.95; (D) estMOI: Polyclonal samples with F_WS_ < 0.95; and (E) F_WS_ distribution of all samples for which Malaria-MOI and estMOI classifications disagreed for polyclonal samples predicted by Malaria-MOI. These panels illustrate the overlap and disagreement between the three methods in classifying sample clonality, highlighting how F_WS_ supports or contrasts with method-specific MOI estimates.
